# Prevalence and determinants of alcohol use among adults living with HIV/AIDS in Ethiopia: a systematic review protocol

**DOI:** 10.1186/s13643-020-01402-w

**Published:** 2020-06-08

**Authors:** Birhanie Mekuriaw, Zelalem Belayneh, Alemayehu Molla, Tsegaye Mehare

**Affiliations:** 1 Department of Psychiatry, College of Health and Medical Science, Dilla University, Dilla, Ethiopia; 2 Department of Biomedical Science, College of Health and Medical Science, Dilla University, Dilla, Ethiopia

**Keywords:** Prevalence, Harmful drinking, Alcohol use, Substance use, Addiction, Drinking habit, HIV/AIDS, Ethiopia

## Abstract

**Background:**

Alcohol use is a challenging problem which attributes to more than 5% of the overall global burden of disease. It is more common among persons with HIV infection than the general population. Although there are separate studies regarding people with HIV/AIDS in Ethiopia, their results are highly variable and discrepant. The objectives of this study will be to evaluate the prevalence of alcohol use and to identify its associated factors among people with HIV/AIDS in Ethiopia.

**Methods:**

A systematic search of electronic databases (from inception onwards) of PubMed/MEDLINE, Embase, PsycINFO, and Cochrane Library will be conducted. Moreover, grey literatures will be searched from different sources (such as Google Scholar, OpenGrey, and World Health Organization websites). Reference lists of the selected articles will also be searched manually. Observational studies (cross-sectional, case-control, cohort) reporting the prevalence of alcohol use and/or its associated factors among adults with HIV/AIDS in Ethiopia will be included. The primary outcomes will be the prevalence of alcohol use among HIV/AIDS population. Secondary outcomes will be the determinants of alcohol use described in the included studies. Two reviewers will independently screen all citations and full-text articles and extract data. The studies’ methodological quality (or bias) will be appraised using an appropriate tool. If feasible, we will conduct a random effects meta-analysis of observational data. Heterogeneity of primary studies will be assessed using the *I*^2^ test. Prevalence estimates will be stratified according to gender, age, and geographical location. Small-study effects (publication bias) also will be examined.

**Discussion:**

Our systematic review and meta-analysis will prevail the pooled prevalence of alcohol use and its determinants among people with HIV/AIDS in Ethiopia. The finding of this study will be helpful to design appropriate preventive and interventional strategies for alcohol use among people with HIV/AIDS. This can have direct or indirect policy responses and clinical implications.

**Systematic review registration:**

PROSPERO CRD42019132524

## Background

Alcohol use is an important public health problem with higher morbidity and mortality rate. According to the World Health Organization (WHO) report, more than 3 million people died each year due to harmful alcohol consumption [1, 2]. Alcohol is considered the third leading cause of death that contributes to 5% of the overall global burden of disease [3]. The morbidity and mortality rates attributed to alcohol are expected to be greater than the combined numbers of deaths due to human immunodeficiency virus (HIV), acquired immunodeficiency syndrome (AIDS), violence, and tuberculosis globally [4, 5].

A bunch of reasons has been identified as contributing factors to the highly prevalent alcohol use problem among people with human immunodeficiency virus/acquired immunodeficiency syndrome (HIV/AIDS) [6, 7]. Some studies showed that medication nonadherence, psychological distress, poor quality of life, family history of alcohol or other substance use, poor coping skills to accept HIV-positive serostatus, and poor social support can contribute to harmful alcohol consumption among people living with HIV/AIDS [8–11].

Despite the high prevalence of alcohol use, there is still a challenge that alcohol or other substance users mostly underreport or totally deny their consumption due to social taboos and its conflict with medical advices. This needs to design strategies with a multi-sectoral effort to elicit the true picture of harmful alcohol consumption which negatively affects the treatment outcomes of HIV/AIDS care [12, 13].

Alcohol use can have negative effects on the treatment outcome, disease progression, and overall quality of life of people with HIV/AIDS [14]. For example, alcohol increases treatment delay, reduces adherence and treatment compliance, fastens disease progression, reduces viral load suppression, and negatively affects the person’s functionality. Individuals with alcohol use problems also often engage in polysubstance use and other risky behaviors like multiple partners and unsafe sex which may in turn result in increased risks of further HIV transmission [15, 16].

In Ethiopia, there are different traditional homemade alcoholic beverages (*Tella*, *Tej*, *Arki*, *Wine*) with different and unestimated alcoholic contents. People commonly use such alcoholic beverages in different parties, ceremonies, and family preparations. In some rural communities of Ethiopia, such homemade traditional alcoholic beverages are consumed on a daily basis, and alcohol is considered as a socially acceptable drink including during their meals [8, 17, 18]. As a result, individuals with HIV/AIDS may use these locally available alcoholic beverages as pain killer, to induce sleep, as a means for social engagement, and to escape from their stressful environment during a crisis.

Although some studies have existed regarding alcohol use among adults with HIV/AID in Ethiopia, there is a great discrepancy and inconsistency of the reported results. For instance, a study conducted in Zewuditu Memorial Hospital showed the lowest prevalence (6.5%) of alcohol use, and the highest prevalence (32.6%) was observed from a study conducted in Jimma [8, 19]. This demonstrates a need for the full picture of the problem to develop effective intervention and for policy responses that can support decreasing alcohol consumption among the HIV/AIDS population. Therefore, the objectives of this systematic review will be to evaluate the prevalence of alcohol use and to identify its associated factors among adults with HIV/AIDS in Ethiopia.

## Methods

This study protocol is being reported in accordance with the reporting guidance provided in the Preferred Reporting Items for Systematic Reviews and Meta-analysis Protocols (PRISMA-P) statement [20, 21] (see Additional file 1). The review protocol has been registered in the International Prospective Register of Systematic Reviews (PROSPERO) [CRD42019132524].

### Information sources and search strategy

A systematic search of PubMed/MEDLINE, Embase, PsycINFO, and Cochrane Library will be conducted from inception onwards. The search strategy will be developed in consultation with a health sciences librarian, which will be reviewed using the Peer Review of Electronic Search Strategies (PRESS) Checklist [22]. The search strategy will be developed in PubMed/MEDLINE (see Additional file 2) and adapted to the other bibliographic databases. Search terms will include subject headings (e.g., MeSH in PubMed/MEDLINE) for each database and free-text words for the key concepts of “prevalence,” “epidemiology,” “alcohol use,” “HIV,” “AIDS,” and “Ethiopia.” Grey or difficult to locate literature will be searched, including Google Scholar, Open Grey, and the World Health Organization (WHO) websites. We will also search reference lists of included studies and related reviews.

### Inclusion criteria

Studies will be selected according to the following criteria: study design, participants, settings, and outcomes of interest.

#### Study design

Observational studies (cross-sectional, case-control, and cohort studies) reporting the prevalence and/or associated factors of alcohol use among adults with HIV/AIDS will be included.

#### Population

Primary studies conducted among adults (age ≥ 18) living with HIV/AIDS will be considered.

#### Settings

Studies conducted in a wide range of HIV people from the community and/or outpatient settings in any Ethiopian region will be considered.

#### Outcomes of interest

The primary outcome will be the prevalence of alcohol use indicating the number of HIV/AIDS people that consume alcohol divided by the HIV/AIDS adult population number at a given point in time. This is often presented as a prevalence (proportion). We will use author-reported definitions according to accepted standardized measurement tools or questionnaires such as Alcohol Use Disorder Identification Test (AUDIT); Cut down, Annoying, Guilt feeling, and Eye Opener (CAGE); or other important measurement tools used to determine the level of alcohol consumption. The secondary outcomes will be the determinants or associated factors of alcohol use reported in the included studies (such as gender, marital status, smoking behavior, mental distress, social support level, and khat chewing habit). No limitations will be imposed on publication status (unpublished studies will be eligible for inclusion) and study conduct period. Only studies published in English (or with an English translation available) will be considered.

### Screening and selection of studies

All articles identified from the literature search will be screened by two reviewers (ZB and BM), independently after duplicate removal by using EndNote. Titles and abstracts of articles returned from the initial searches will be screened based on the eligibility criteria outlined above (the “Inclusion criteria” section). Full texts will be examined in detail and screened for eligibility. References of all considered articles will be hand-searched to identify any relevant report missed in the search strategy. Any disagreements will be resolved by a discussion, if necessary. A PRISMA flow chart showing details of studies included and excluded at each stage of the study selection process will be provided.

### Data abstraction

A data extraction form will be designed in Microsoft Excel and used by two reviewers (BM and ZB) to extract equivalent information from each study report. A single row will be used for each study. Information of interest will include the following.

#### Study characteristics

The following are the study characteristics: name of the first author, study design, year of publication, journal, year (or period) of study conduct, sample size, setting, geographical location where the study is conducted, and other fields to capture data relevant to the assessment of study methodological quality.

#### Participant characteristics

The following are the participant characteristics: population sampled, age (e.g., mean with standard deviation, range), and gender (e.g., percentage of female and male participants).

#### Outcome results

Definitions and measures used to make diagnosis (of alcohol use and the determinants of use), prevalence estimates (e.g., number of subjects with the outcome, proportion, and 95% confidence interval), and any prevalence estimates stratified by age, gender, or study location. For the second outcome (determinants of alcohol use), important data will be extracted using two by two tables for each predictor. Any disagreement between the two authors during data extraction will be solved through a discussion.

### Quality assessment

Two authors will independently assess the overall qualities of primary articles using the Newcastle-Ottawa Scale quality assessment tool [23, 24]. The tool has three different sections. The first section assesses the methodological quality of each study, the second part examines the comparability of primary studies, and the last part assesses articles with respect to their statistical analysis and interpretation. Any disagreements between the two assessors are expected to be negotiated through a discussion and by taking the average score of the two different assessment results. Articles fulfilling equal to or greater than 50% of the quality assessment criteria score will be included in this review.

### Data synthesis

The data from each study will be used to build evidence tables of an overall description of the included studies. Crude prevalence estimates (number of cases/sample size) will be presented along with 95% confidence intervals. If feasible and appropriate, prevalence data points from primary studies will be used to perform random effects meta-analyses. Since heterogeneity is expected, we will estimate the pooled prevalence and its 95% confidence interval using the random effects model. The random effects model [25] assumes the study prevalence estimates follow a normal distribution, considering both within-study and between-study variations. We will also conduct a quality-effects model to examine how the quality of each study changed the pooled estimate compared with the results from the random-effects meta-analysis [26]. Forest plots will be used to visualize pooled estimates and the extent of heterogeneity among studies.

We will quantify statistical heterogeneity by estimating the variance between studies using the *I*^2^ statistic [27]. The *I*^2^ statistic is the proportion of variation in prevalence estimates that is due to the genuine variation in prevalence rather than sampling (random) error. The *I*^2^ statistic ranges between 0 and 100% (with values of 0–25% and 75–100% taken to indicate low and considerable heterogeneity, respectively). We will also report the Cochran *Q* test with a *p* value of < 0.05 considered statistically significant. All statistical analyses will be performed using Stata-14 (StataCorp LP, College Station, TX, USA)

If sufficient studies are identified and data points are available, potential sources of heterogeneity will be investigated further by subgroup analyses according to baseline characteristics and methodological covariates. We plan to conduct analyses by gender (male vs. female), geographical location of studies (e.g., using regions where the studies are conducted), study quality (e.g., low vs. moderate/high quality), and publication year.

Small study effects (publication bias across studies) will be assessed by inspection of the funnel plots for asymmetry and Egger’s test [28–30], with the results considered to indicate potential small study effects when *p* values are < 0.10.

## Discussion

This systematic review and meta-analysis will determine the prevalence of alcohol use among HIV/AIDS patients in Ethiopia. In addition, this study will identify factors associated with alcohol use among people with HIV/AIDS. Even though there are empirical evidences about the magnitude of alcohol use and preventive measures, it remains still a challenging problem in the management of people with HIV/AIDS. The findings of this review could be an important input in health policy and planning debates, such as the development of evidence-based prevention and intervention programs for people with HIV/AIDS. This review will identify and fill the knowledge gaps in the field with new research. On this regard, implications for future observational studies will be discussed.

The proposed systematic review and meta-analysis will be reported in accordance with the reporting guidance provided in the Preferred Reporting Items for Systematic Reviews and Meta-analyses (PRISMA) statement [20, 21] and the Meta-analysis Of Observational Studies in Epidemiology (MOOSE) reporting guideline [31]. Any amendments made to this protocol during the study conduct will be reported in the final manuscript and reported in PROSPERO. Results will be disseminated through publication in a peer-reviewed journal.

There are several limitations to our planned systematic review. We anticipate identifying observational studies conducted in Ethiopia with different study designs, populations, and contexts and with a variable quality of reporting of methods and results. In addition, publication and reporting biases can distort the planned review and meta-analysis.

## Supplementary information

**Additional file 1.** PRISMA-P Checklist.

**Additional file 2.** Search strategy for PubMed.

## Data Availability

Not applicable.

## Abbreviations

HIV/AIDS: Human immunodeficiency virus /acquired immunodeficiency syndrome
MOOSE: Meta-analysis Of Observational Studies in Epidemiology
PRESS: Peer Review of Electronic Search Strategies
PRISMA-P: Preferred Reporting Items for Systematic Reviews and Meta-analysis
WHO: World Health Organization
