# Supplementary material for: Prevalence and determinants of alcohol use among adults living with HIV/AIDS in Ethiopia: a systematic review protocol
Source: Syst Rev. 2020 Jun 8;9:138. doi: 10.1186/s13643-020-01402-w (PMC7282110; doi:10.1186/s13643-020-01402-w)
Supplement: Supplementary file 2 — Additional file 2. Search strategy for PubMed. [file 13643_2020_1402_MOESM2_ESM.docx]

| Search | Query |
| --- | --- |
| #1 | ("Underage Drinking"[Mesh]) OR ( "Alcohol Drinking"[Mesh] OR "Alcoholism"[Mesh] ) |
| #2 | Alcohol use[tw] |
| #3 | "Acquired Immunodeficiency Syndrome"[Mesh] OR "AIDS-Related Complex"[Mesh] OR "HIV Seropositivity"[Mesh] OR "HIV"[Mesh] OR "AIDS-Related Opportunistic Infections"[Mesh] OR "AIDS Arteritis, Central Nervous System"[Mesh] OR "AIDS-Associated Nephropathy"[Mesh] OR "AIDS Dementia Complex"[Mesh] OR "AIDS Serodiagnosis"[Mesh] |
| #4 | HIV\AIDS[tw] |
| #5 | "Ethiopia"[Mesh] OR "hemoglobin Ethiopia" [Supplementary Concept] |
| #6 | Ethiopia[tw] |
| #7 | (#1) OR #2 |
| #8 | (#3) OR #4 |
| #9 | (#5) OR #6 |
| #10 | ((#7) AND #8) AND #9 |
| #11 | (#10) AND (humans [MeSH Terms] AND English [lang]) |

Additional file-2: Search strategy for PubMed
